# Efficacy and safety of guanxinshutong capsule combined with western medicine on stable angina pectoris: a systematic review and meta-analysis

**DOI:** 10.3389/fphar.2024.1444388

**Published:** 2024-10-30

**Authors:** Liyuan Yu, Lulu Wu, Weihang Peng, Peiying Huang, Li Chen, Yi Deng, Meida Wang, Jing Zeng, Bojun Chen

**Affiliations:** ^1^ The Second Clinical Medical School of Guangzhou University of Chinese Medicine, Guangzhou, China; ^2^ Guangdong Provincial Key Laboratory of Research on Emergency in Traditional Chinese Medicine, Clinical Research Team of Prevention and Treatment of Cardiac Emergencies with Traditional Chinese Medicine, Guangzhou, China; ^3^ Emergency Department of Guangdong Provincial Hospital of Traditional Chinese Medicine, Guangzhou, China

**Keywords:** stable angina pectoris, guanxinshutong capsule, efficacy, safety, meta-analysis

## Abstract

**Aims:** To systematically evaluate the efficacy and safety of the Guanxinshutong capsule (GXST) combined with Western medicine (WM) in treating stable angina pectoris (SAP).

**Methods:** Randomized controlled trials (RCTs) evaluating the efficacy of GXST combined with WM for the treatment of patients with SAP were searched across several databases, including the Cochrane Library, PubMed, Embase, the Chinese National Knowledge Infrastructure (CNKI), the Chinese Science and Technology Journal Database (VIP), and Wan Fang, from inception until 30 April 2024. Two independent reviewers rigorously performed study selection, data extraction, and quality assessment. Version 2 of the Cochrane risk-of-bias tool for randomized trials (RoB 2) was employed to assess the methodological quality of included RCTs. R version 4.2.2 was applied for data synthesis.

**Results:** Between 2012 and 2024, 31 RCTs involving 4,172 patients were identified, with 2,101 in the experimental group and 2,071 in the control group. GXST and WM combination was significantly more effective than WM alone across several metrics: clinical efficacy rate (odds ratio [OR] = 4.05, 95% confidence interval [CI] = 3.42–4.80), electrocardiogram improvement (OR = 3.39, 95% CI = 2.35–4.87), enhancement in left ventricular ejection fraction (mean difference [MD] = 1.07, 95% CI = 0.69–1.46), reduction in total cholesterol levels (MD = −0.78, 95% CI = −1.20 to −0.35), decrease in tumor necrosis factor-alpha (MD = −1.36, 95% CI = −2.18 to −0.53), and improvement in Chinese medicine evidence score (OR = 3.77, 95% CI = 2.20–6.43). No significant difference was observed in the reduction in C-reactive protein levels (MD = −6.66, 95% CI = −15.91 to 2.59), triglyceride levels (MD = −1.62, 95% CI = −3.39 to 0.15), or in the occurrence of adverse drug reactions (OR = 0.60, 95% CI = 0.23–1.57). Based on meta-regression and subgroup analyses, the observed heterogeneity was attributed to variations in GXST capsule dosage, the duration of treatment, and the baseline characteristics of patients.

**Conclusion:** GXST and WM combination therapy demonstrates the potential to enhance clinical outcomes for SAP patients. Nevertheless, additional rigorous studies are imperative to substantiate the reliability and safety of this combined treatment modality.

**Systematic Review Registration:** The protocols for this meta-analysis were registered in the International Prospective Register of Systematic Reviews (PROSPERO, https://www.crd.york.ac.uk/PROSPERO/display_record.php?RecordID=543537, Identifier CRD42024543537).

## 1 Introduction

Stable angina pectoris (SAP) is a manifestation of coronary heart disease (CHD) (Karabağ et al., 2018). The underlying mechanism involves severe stenosis and obstruction of coronary arteries, resulting in an imbalance between coronary blood flow and myocardial demand (Ford et al., 2018). This imbalance, often due to increased cardiac load, leads to acute ischemia and hypoxia of the myocardium (Gunata and Parlakpinar, 2021). Symptoms present as transient discomfort in the posterior sternum and precordial area, characterized by short-lived compressive pain, heaviness, or suffocation (i.e., angina pectoris (Gunata and Parlakpinar, 2021)). These symptoms can be triggered by factors such as exertion, emotional stress, and overeating (Joshi and de Lemos, 2021). Typically, the symptoms subside within minutes after rest or administration of nitrates (Shao et al., 2020). Additionally, among non-communicable diseases (NCDs), cardiovascular diseases (CVDs) are a major contributor to the disease burden and the leading cause of death worldwide (2020).

The treatment priorities for SAP are to alleviate symptoms, improve quality of life, and prevent myocardial infarction and death (Feng et al., 2023). First-line medications include nitrates, β-blockers, calcium channel blockers, and antiplatelet agents (Liu et al., 2022). These medications belong to a category of chemically synthesized drugs and are not derived from natural plants; therefore, they are commonly referred to as Western medicine (WM). These drugs aim to achieve several therapeutic goals, including reducing myocardial oxygen demand and heart workload, improving blood flow, and preventing thrombus formation. Nitrates can relax vascular smooth muscle and reduce heart preload, thereby decreasing myocardial oxygen demand and alleviating angina symptoms (Patra et al., 2023; Singh et al., 2024). β-Blockers have been shown to reduce heart rate, blood pressure, and contractility, thereby decreasing myocardial oxygen demand and alleviating angina symptoms (Palatini et al., 2024). In low-risk populations, aspirin, used for primary prevention, has been shown to reduce the risk of non-fatal myocardial infarction, non-fatal stroke, and vascular death (Judge et al., 2020). However, these WM therapies have some drawbacks. For example, β-blockers are associated with bradycardia and atrioventricular block (Lu et al., 2016); nitrate drugs may cause hypotension, increased intracranial pressure, dizziness, and headache (Londono-Hoyos et al., 2018; Rivasi et al., 2020); and antiplatelet agents may lead to severe bleeding and gastrointestinal adverse reactions (Benamouzig et al., 2022), with the risk of bleeding potentially outweighing the benefits. For patients with severe CHD who are inadequately controlled with medication, percutaneous coronary intervention (PCI) or coronary artery bypass grafting (CABG) may be considered (Xie et al., 2021). While PCI and CABG can effectively improve blood flow and alleviate angina symptoms, they also pose risks of postoperative complications such as thrombus formation, myocardial infarction, and heart failure (Beerkens et al., 2022). Moreover, these surgeries are often characterized by a long recovery period, which significantly reduces the quality of life of patients. Furthermore, continuous medical treatment and monitoring are essential due to the chronic nature of SAP. Therefore, developing new adjunctive medications is crucial to address these limitations, improve the quality of life, and enhance treatment adherence among patients with SAP.

Guanxinshutong capsule (GXST) is a traditional Chinese medicine (TCM) that possesses therapeutic effects such as promoting blood circulation to remove blood stasis, activating meridians and collaterals, and promoting the flow of qi to relieve pain (Li et al., 2019). It is clinically used for the treatment of CHD, acute myocardial infarction, angina pectoris, and other diseases (Wang et al., 2021). This capsule is composed of five distinct traditional Chinese medicines (TCMs), including *Salvia miltiorrhiza* Bunge [Lamiaceae, salviae miltiorrhizae radix et rhizoma], *Choerospondias axillaris* (Roxb.) [Anacardiaceae, Choerospondiatis Fructus], *Syzygium aromaticum* (L.) [Myrtaceae, Caryophylliflos], *Cinnamomum camphora* (L.) Presl [Lauraceae, Borneolum], and *Cephalostachyum chinense* (Rendle) [Poaceae, Bambusae Concretio Silicea]. In the Traditional Chinese Medicine Systems Pharmacology Database and Analysis Platform (TCMSP, http://tcmspw.com/tcmsp.php), the screening criteria were established as Dyslipidemia (DL) ≥0.18, Obesity (OB) ≥40%, Cancer Colon 2 (Caco-2) ≥−0.4, and Hyperlipidemia (HL) ≥4 (Zhang et al., 2020), and the botanical drug ingredients of GXST were queried. The composition is detailed in Supplementary File S1.

Previous clinical studies have shown that the addition of GXST to conventional treatment can help alleviate angina symptoms in patients with SAP and improve their quality of life (Wang et al., 2021). Through its effects of promoting blood circulation to remove blood stasis and activating meridians and collaterals, GXST effectively improves coronary blood circulation, increases coronary blood flow, and reduces the degree of myocardial ischemia and hypoxia (Zhang et al., 2021). It can also provide comprehensive health protection for patients by improving blood circulation, relieving pain, and preventing further deterioration of the disease (Wang et al., 2020). Two previous meta-analyses (Sui et al., 2016; Jia and Wei, 2017) have shown that GXST and WM combination therapy can improve the treatment efficacy and electrocardiogram (ECG) performance of SAP patients. However, there is currently no comprehensive systematic evaluation and safety analysis of the therapeutic effects of this combination therapy, including the improvement of cardiac function, laboratory indicators, and TCM syndrome scores. Moreover, the two previous meta-analyses were published 5 years ago, and the number of included studies was relatively small, with one meta-analysis including 12 studies (Sui et al., 2016) and the other 7 studies (Jia and Wei, 2017). The present meta-analysis has included more up-to-date studies, thus enhancing the reliability and generalizability of the results. Additionally, we have considered more outcome indicators to comprehensively evaluate the efficacy of GXST and WM combination therapy in treating patients with SAP, providing a reference for clinical practice and future research.

## 2 Materials and methods

This study was conducted according to the Preferred Reporting Items for Systematic Reviews and Meta-Analyses (PRISMA) Extension Statement and was registered with the International Prospective Register of Systematic Reviews (PROSPERO, https://www.crd.york.ac.uk/PROSPERO/display_record.php?RecordID=543537, registration number CRD42024543537). A PRISMA checklist is detailed in Supplementary File S2.

### 2.1 Search strategy

A comprehensive search for randomized controlled trials (RCTs) evaluating the efficacy of GXST and WM combination therapy in treating patients with SAP was conducted across seven databases, including PubMed, Embase, Cochrane Library, Chinese National Knowledge Infrastructure (CNKI), Wan Fang Database, Chinese Science and Technology Journal Database (VIP), and Chinese Biomedical Literature Database. The search spanned from the inception of each database until 30 April 2024. Search terms in English encompassed “GuanxinShutong capsule” and “stable angina pectoris”. The search methodology applied is delineated in Supplementary File S3.

### 2.2 Inclusion and exclusion criteria

The inclusion criteria were as follows.

(1) Study subjects: Patients with SAP.
(2) Control group treatment: The control group received WM treatments, including nitrates, β-blockers, calcium channel blockers, and antiplatelet agents.
(3) Experimental group treatment: The experimental group received a combination of GXST intervention and the same WM treatments as the control group.
(4) Study Design: RCTs.
(5) Selected study outcomes: Studies were considered if any of the following outcomes occurred.

Primary outcomes.

(1) Effective clinical rate: This was defined as the percentage of patients who showed improvement after treatment. The effective clinical rate was calculated by subtracting the number of ineffective cases from the total number of cases and dividing the result by the total number of cases. The assessment of treatment efficacy was based on angina symptoms. Significant efficacy was defined as substantial alleviation of symptoms and signs after therapy. An effective outcome referred to an improvement in symptoms and signs after treatment, whereas an ineffective outcome denoted no significant change or a deterioration in symptoms and signs after treatment.
(2) Effective ECG rate: This was defined as the percentage of patients who showed improved myocardial ischemia, indicated by changes in their ECGs, such as the normalization of T-wave inversion.

Secondary outcomes.

(1) Changes in left ventricular ejection fraction (LVEF) and C-reactive protein (CRP), tumor necrosis factor-alpha (TNF-α), total cholesterol (TC), and triglyceride (TG) levels were observed before and after treatment.
(2) Chinese medicine evidence score: The Chinese medicine evidence score was calculated according to the Guidelines for Clinical Research of New Chinese Medicines (GCRNCM). Symptoms of chest tightness, shortness of breath, palpitation, and chest pain were observed in both groups and categorized into 2 (mild), 4 (moderate), and 6 (severe) points according to GCRNCM. Significant efficacy was defined as the disappearance of pre-treatment symptoms and reduction in the total score by >80%; an effective outcome was defined as the relief of pre-treatment symptoms and reduction in the total score by about 40%–79%; and an ineffective outcome was defined as insignificant relief of pre-treatment symptoms and reduction in the total score by <40%.
(3) Adverse drug reactions (ADRs).

The exclusion criteria were as follows.

(1) Incomplete or significantly erroneous outcome data.
(2) Patients with other severe CVDs such as acute myocardial infarction or heart failure.
(3) Studies showing randomization failure or significant baseline differences between groups.
(4) The experimental or control group received other TCMs or herbal treatments.

### 2.3 Data extraction

A comprehensive approach integrating software and manual methods was employed to identify relevant studies. Initially, all duplicate studies were rigorously eliminated. Subsequently, two reviewers independently screened titles and abstracts, adhering strictly to predefined inclusion and exclusion criteria, followed by a thorough review of the full text of the selected articles and data extraction. The extracted data encompassed the first author’s name, publication year, sample size, age range, specific disease progression metrics, treatment duration, and dosage. Outcome indicators and pertinent quality assessment information were also extracted. The obtained results were cross-verified to ensure precision and accuracy. Any discrepancies were resolved through consensus discussions between the two reviewers, or with a third party, if necessary.

### 2.4 Analysis of study quality

The risk of bias in the included RCTs was rigorously assessed using version 2 of the Cochrane Collaboration’s risk-of-bias tool for randomized trials (RoB 2) (Sterne et al., 2019), as outlined by Sterne et al. (2019). This instrument scrutinized five pivotal domains: the randomization process, deviations from intended interventions, missing outcome data, measurement of the outcome, and the selection of the reported result. Each domain was categorized as having “low-risk,” “high-risk,” or “some concerns” of bias. A trial was considered to have an overall ‘low risk’ of bias only if all domains were unanimously classified as having a ‘low risk’. Any discrepancies were resolved through consensus discussions between two reviewers, or with a third party, if necessary, to ensure accuracy and consistency.

### 2.5 Data analysis

All statistical analyses were performed using R statistical software (version 4.2.2) and its Meta package (version 6.5.0). Continuous and categorical variables were evaluated using the mean difference (MD) and odds ratio (OR), respectively, along with a 95% confidence interval (95% CI). The statistical significance was set at *p* < 0.05. Heterogeneity within each study was assessed using the Q statistic and I^2^ test. Forest and Labbé plots were employed to visually inspect heterogeneity and identify variation sources. The I^2^ test was also applied to evaluate heterogeneity during data integration. When I^2^ was less than 50%, indicating low heterogeneity, a fixed-effects model was applied. Conversely, a random-effects model was applied when I^2^ exceeded 50%, suggesting high heterogeneity. To address potential heterogeneity and ensure the robustness of the results, meta-regression and sensitivity analyses were performed using the “metareg” and “metainf” commands for all outcome indicators with I^2^ values ≥ 50%. In addition, subgroup analyses were conducted based on positive covariates identified in the meta-regression. Sensitivity analyses were performed by excluding each literature individually. For outcomes involving more than five studies, potential publication bias was explored using adjusted funnel plots and Egger’s and Begg’s tests, implemented via the “metabias” command.

## 3 Results

### 3.1 Literature retrieval and study characteristics

**FIGURE 1:**
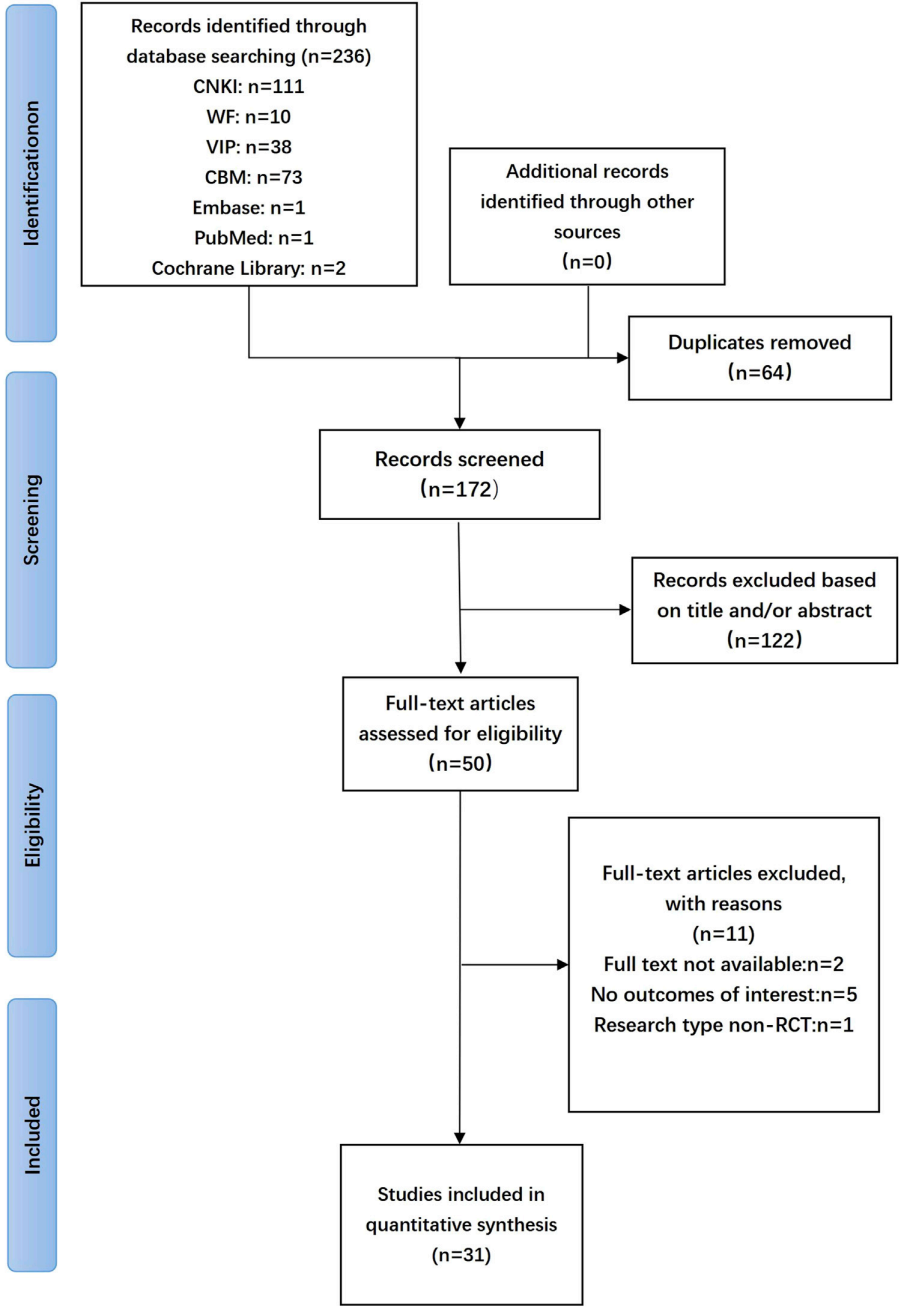
Flow diagram of systematic literature search.

An initial retrieval yielded 236 studies, of which 31 articles (Li and Li, 2012; Wang et al., 2012; Cao and Yue, 2013; Jiang, 2013; Liang et al., 2013; Liu, 2013; Yu et al., 2013; Qi, 2015; Wang and Wu, 2015; Wu et al., 2015; Zhao et al., 2015; An, 2016; Peng, 2016; Ji, 2017; Ren et al., 2017; Yu, 2017; Zhu and Liu, 2017; Geng et al., 2018; Shi and Abudujilili, 2018; Yang and Zhang, 2018; Li, 2019; Li et al., 2019; Liu et al., 2019; Wan et al., 2019; Luo, 2021; Wang, 2021; You, 2021; Cai, 2022; Pan et al., 2022; Shi, 2022; Jia and Li, 2023) were included in the final analysis. The flowchart of the literature search and screening process is depicted in Figure 1. The characteristics of eligible studies are outlined in Table 1.

**TABLE 1:** Characteristics of the included studies.

Study ID	Sample size (M/F)		Age (years)		Course of disease (years)		Interventions		
	T	C	T	C	T	C	T	C	
Li et al. (2019)	143 (73/70)	144 (82/62)	56.6 ± 8.1	55.3 ± 8.3	NR	NR	GXST + WM	WM	Aspirin Atorvastatin ACEI/ARB β-receptor blocker CCB
Ji (2017)	43 (20/23)	42 (24/18)	45.12 ± 0.61	46.12 ± 0.41	1–5	1–6	GXST + WM	WM	Aspirin 100 mg po, qd Atorvastatin 10 mg po, qd ISDN 10 mg po, qd
Ren et al. (2017)	41 (25/16)	40 (23/17)	66.05 ± 8.48	65.48 ± 8.37	NR	NR	GXST + WM	WM	Aspirin 100 mg po, qd Atorvastatin 10 mg po, qd Metoprolol 90–190 mg po,qd
Wan et al. (2019)	200(NR)	200(NR)	NR	NR	NR	NR	GXST + WM	WM	NR
Liang et al. (2013)	40 (27/13)	40 (29/11)	38–83	40–84	6–24	7–24	GXST + WM	WM	Aspirin Atorvastatin ACEI Metoprolol
Yang and Zhang (2018)	30 (19/11)	30 (18/12)	65.45 ± 1.23	66.45 ± 1.98	4.12 ± 1.34	4.23 ± 1.05	GXST + WM	WM	ISDN 10 mg po, qd Aspirin 100 mg po, qd Atorvastatin 10 mg po, qd
Wang (2021)	40 (24/16)	40 (22/18)	43.42 ± 7.39	43.68 ± 7.45	4.41 ± 1.16	4.58 ± 1.24	GXST + WM	WM	ISDN 20 mg po, tid
You (2021)	30 (16/14)	30 (18/12)	57.43 ± 3.34	56.52 ± 3.51	NR	NR	GXST + WM	WM	ISDN 30 mg po,tid
Jia and Li (2023)	57 (31/26)	57 (34/23)	60.05 ± 5.91	59.42 ± 6.28	0.02–0.06	0.02–0.07	GXST + WM	WM	Fluvastatin 40 mg po, qd
Shi (2022)	48 (26/22)	48 (25/23)	66.18 ± 1.25	62.97 ± 1.20	2.78 ± 1.01	2.90 ± 1.05	GXST + WM	WM	Aspirin Atorvastatin Metoprolol 6.25 mg po,bid
Liu et al. (2019)	66 (37/29)	66 (39/27)	69.5 ± 5.7	69.6 ± 5.2	2.3 ± 0.2	2.1 ± 0.3	GXST + WM	WM	Aspirin Atorvastatin Metoprolol 6.25 mg po,bid
Pan et al. (2022)	54 (31/23)	54 (33/21)	58.69 ± 4.18	57.92 ± 3.96	3.14 ± 0.28	3.19 ± 0.32	GXST + WM	WM	Aspirin β-receptor blocker nicorandil
Luo S (2021)	150 (68/82)	150 (66/84)	58.7 ± 3.9	59.4 ± 3.5	NR	NR	GXST + WM	WM	simvastatin 20 mg po, qn ISDN 20 mg po, qd Clopidogrel 75 mg po,qd
Wu et al. (2015)	181 (114/67)	181 (100/81)	63.0 ± 1.0	62.5 ± 1.0	6.11 ± 1.67	6.01 ± 1.71	GXST + WM	WM	ISDN 20 mg po, bid Aspirin 100 mg po, qd Atorvastatin 10 mg po, qd amlodipine 5 mg po, qd
Yu (2017)	31 (17/14)	31 (15/16)	64–76	65–78	0.5–14	0.6–15	GXST + WM	WM	Trimetazidine Aspirin Atorvastatin ISDN
An (2016)	45 (20/25)	45 (22/23)	58.3 ± 12.3	57.6 ± 10.9	5.7 ± 1.4	6.4 ± 1.8	GXST + WM	WM	bisoprolol 2.5 mg po, qd ISDN 20 mg po, bid Aspirin 100 mg po, qd Atorvastatin 10 mg po, qd
Wang et al. (2012)	150 (89/61)	130 (67/63)	55 ± 10	58 ± 11	5	4.5	GXST + WM	WM	ISDN 5 mg po, tid
Qi (2015)	40 (22/18)	40 (24/16)	45.1 ± 2.9	45.0 ± 3.5	7.2 ± 3.1	6.9 ± 4.0	GXST + WM	WM	ISDN Diltiazem 90 mg po, qd
Yu et	40 (25/15)	40 (27/13)	63.40 ± 5.24	65.25 ± 5.40	0.17–25	0.25–24	GXST + WM	WM	NR
Li and Li (2012)	96 (42/54)	92 (40/52)	57	55.6	0.5–1.5	0.42–1.5	GXST + WM	WM	NR
Shi and Abudujilili (2018)	41 (21/20)	41 (20/21)	60.4 ± 11.1	59.2 ± 12.4	6.8 ± 3.6	7.1 ± 3.8	GXST + WM	WM	ISDN Aspirin Atorvastatin β-receptor blocker
Liu (2013)	43 (26/17)	40 (27/13)	60.8 ± 5.7	61.1 ± 7.2	NR	NR	GXST + WM	WM	ISDN 10 mg po, qd Aspirin 100 mg po, qd Atorvastatin 10 mg po, qd
Peng (2016)	30(NR)	30(NR)	61.2 ± 3.4	61.2 ± 3.4	7.1 ± 2.5	7.1 ± 2.5	GXST + WM	WM	Aspirin 100 mg po, qd Atorvastatin 10 mg po, qd amlodipine 5 mg po, qd
Wang and Wu (2015)	40 (26/14)	40 (27/13)	59.9 ± 7.4	60.4 ± 7.6	0.25–20	0.5–22	GXST + WM	WM	Aspirin Atorvastatin ISDN
Cao and Yue (2013)	42 (29/13)	42 (31/11)	61.3 ± 5.2	60.1 ± 6.0	7.8 ± 3.7	7.2 ± 4.3	GXST + WM	WM	ISDN Diltiazem 90 mg po, qd
Zhao et al. (2015)	67(NR)	67(NR)	NR	NR	NR	NR	GXST + WM	WM	Aspirin 100 mg po, qd Atorvastatin 20 mg po, qd ISDN 20 mg po, bid
Geng et al. (2018)	60 (31/29)	60 (32/28)	18–68	19–70	2–6	1–5	GXST + WM	WM	NR
Cai (2022)	62 (43/19)	62 (41/21)	62.69 ± 4.80	62.63 ± 4.7	NR	NR	GXST + WM	WM	ISDN+15%GS 20 mg + 500 mL ivgtt, qd amlodipine 10 mg po, qd
Li et al. (2019)	60 (37/23)	60 (40/20)	53.4 ± 2.1	52.6 ± 1.7	4.8 ± 2.2	5.3 ± 1.5	GXST + WM	WM	β-receptor blocker ISDN+15%GS 20 mg + 500 mL ivgtt, qd amlodipine 10 mg po, qd
Zhu ang Liu (2017)	65 (38/27)	65 (40/25)	52.8 ± 2.4	52.4 ± 1.8	4.9 ± 2.1	4.7 ± 1.5	GXST + WM	WM	β-receptor blocker ISDN+15%GS 20 mg + 500 mL ivgtt, qd amlodipine 10 mg po, qd
Jiang (2013)	66 (38/28)	66 (40/26)	40–71	40–71	NR	NR	GXST + WM	WM	β-receptor blocker ISDN+15%GS 20 mg + 500 mL ivgtt, qd amlodipine 10 mg po, qd

**Abbreviations: T:** treatment group; **C:** control group; **M:** males; F: females; **GXST:** guanxinshutong capsule; **WM:** western medication; NR: not reported.

### 3.2 Analysis of study quality

**FIGURE 2:**
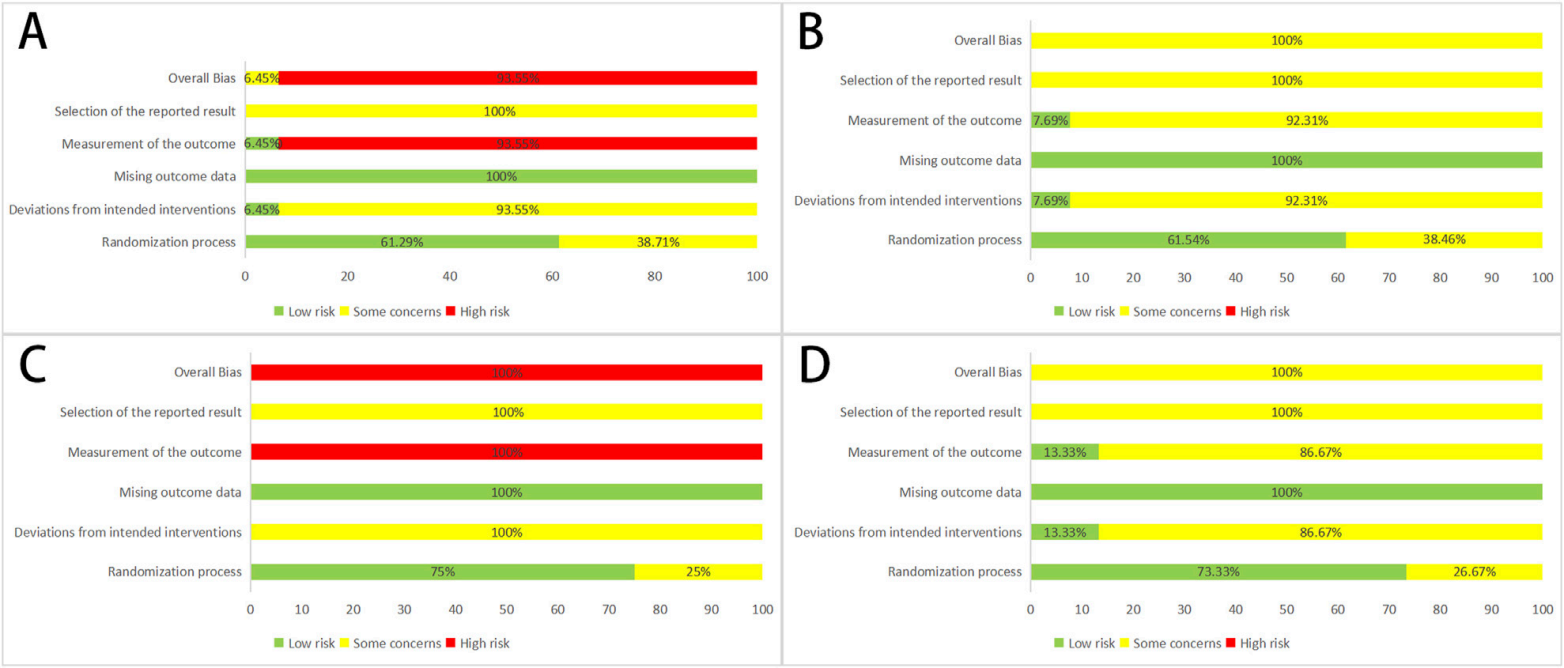
Assessment of risk bias **(A)** Effective clinical rate; **(B)** Effective rate in electrocardiogram; **(C)** Chinese medicine evidence score; **(D)** ARDs; ARDs, adverse medicine events.

Of the included studies, 14 RCTs (45.16%) (Cao and Yue, 2013; Yu et al., 2013; Wang and Wu, 2015; An, 2016; Ren et al., 2017; Zhu and Liu, 2017; Shi and Abudujilili, 2018; Li, 2019; Luo, 2021; Wang, 2021; You, 2021; Pan et al., 2022; Shi, 2022; Jia and Li, 2023) used the randomized table of numbers method, 2 RCTs (6.45%) (Zhao et al., 2015; Li et al., 2019) used envelope sampling, and 1 RCT (3.23%) (Wu et al., 2015) used random lotteries. The remaining 14 RCTs (45.16%) (Li and Li, 2012; Wang et al., 2012; Jiang, 2013; Liang et al., 2013; Liu, 2013; Qi, 2015; Peng, 2016; Ji, 2017; Yu, 2017; Geng et al., 2018; Yang and Zhang, 2018; Liu et al., 2019; Wan et al., 2019; Cai, 2022) did not state the specific randomization method, which might introduce uncertainties in the study results. None of the studies provided an exhaustive explanation of allocation concealment, raising “some concerns” about the randomization process. Only 2 RCTs (6.45%) (Wang et al., 2012; Li et al., 2019) reported using double-blind methods; none of the other studies mentioned blinding. None of the trials clearly described pre-designed procedures or conducted adequate analyses to assess the effects of intervention allocation. This led to “some concerns” about the “selection of the reported result” and “deviations from intended interventions”. Since all outcomes were assessed based on a specific number of patients, the likelihood of bias due to missing outcome data is minimal. However, clinical efficiency and the Chinese medicine evidence score were considered “high risk” for “measurement of the outcome” due to their subjective nature. Therefore, the studies were considered “high risk' for ‘overall bias” (Figure 2).

### 3.3 Meta-analysis results

#### 3.3.1 Primary outcomes

##### 3.3.1.1 Effective clinical rate

**FIGURE 3:**
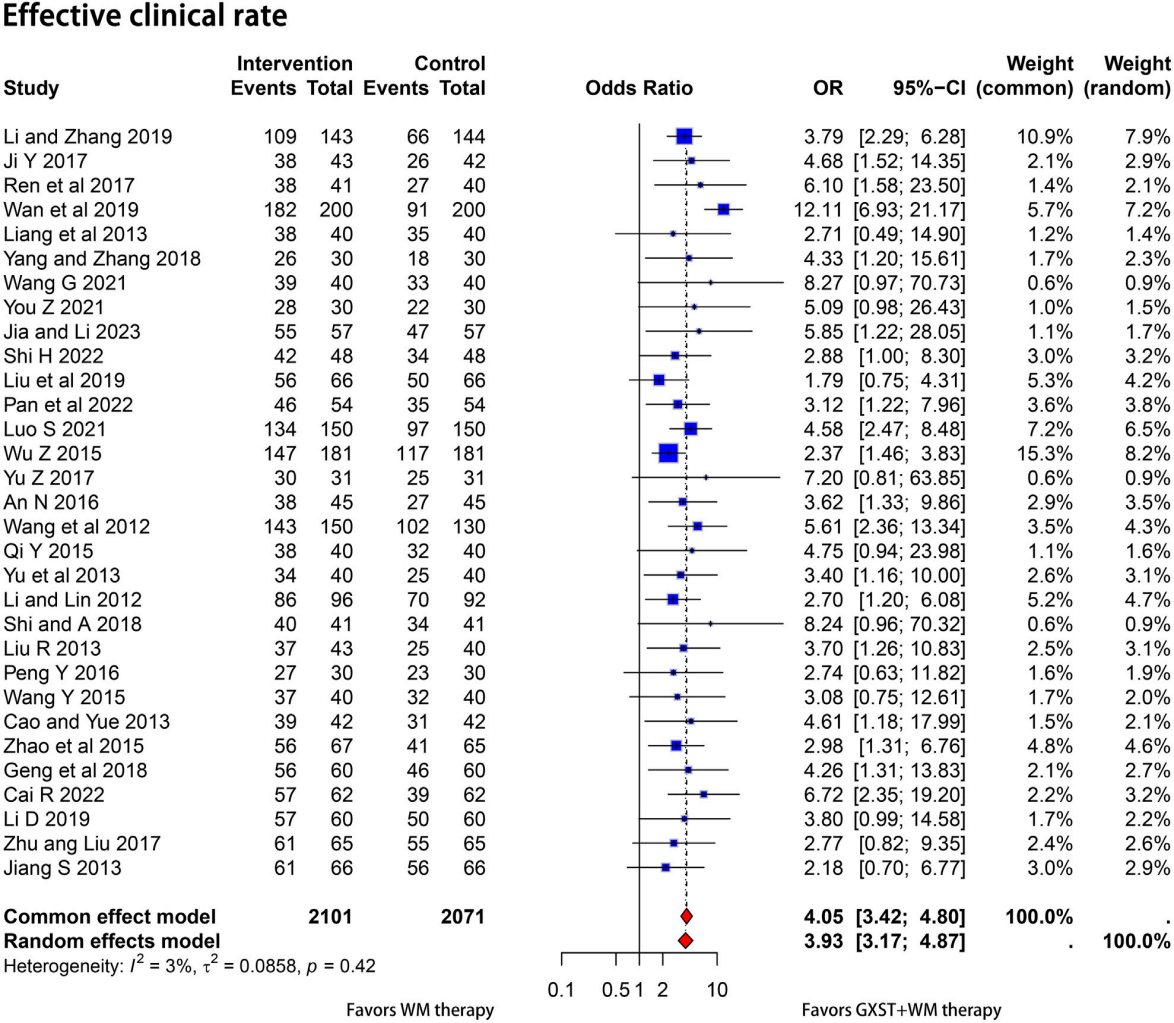
Forest plot for the meta-analysis. Effective clinical rate; GXST, Guanxinshutong; WM, Western medicine.

The efficacy was evaluated based on the improvement of clinical symptoms. Thirty-one studies (Li and Li, 2012; Wang et al., 2012; Cao and Yue, 2013; Jiang, 2013; Liang et al., 2013; Liu, 2013; Yu et al., 2013; Qi, 2015; Wang and Wu, 2015; Wu et al., 2015; Zhao et al., 2015; An, 2016; Peng, 2016; Ji, 2017; Ren et al., 2017; Yu, 2017; Zhu and Liu, 2017; Geng et al., 2018; Shi and Abudujilili, 2018; Yang and Zhang, 2018; Li, 2019; Li et al., 2019; Liu et al., 2019; Wan et al., 2019; Luo, 2021; Wang, 2021; You, 2021; Cai, 2022; Pan et al., 2022; Shi, 2022; Jia and Li, 2023) discussed clinical efficacy. Since homogeneity was found between the studies (*p* = 0.42, I^2^ = 3%), the fixed-effects model was utilized, and the results demonstrated that the experimental group exhibited a significant improvement in patients’ clinical symptoms (OR = 4.05, 95% CI = 3.42–4.80) (Figure 3).

##### 3.3.1.2 Effective rate in ECG

**FIGURE 4:**
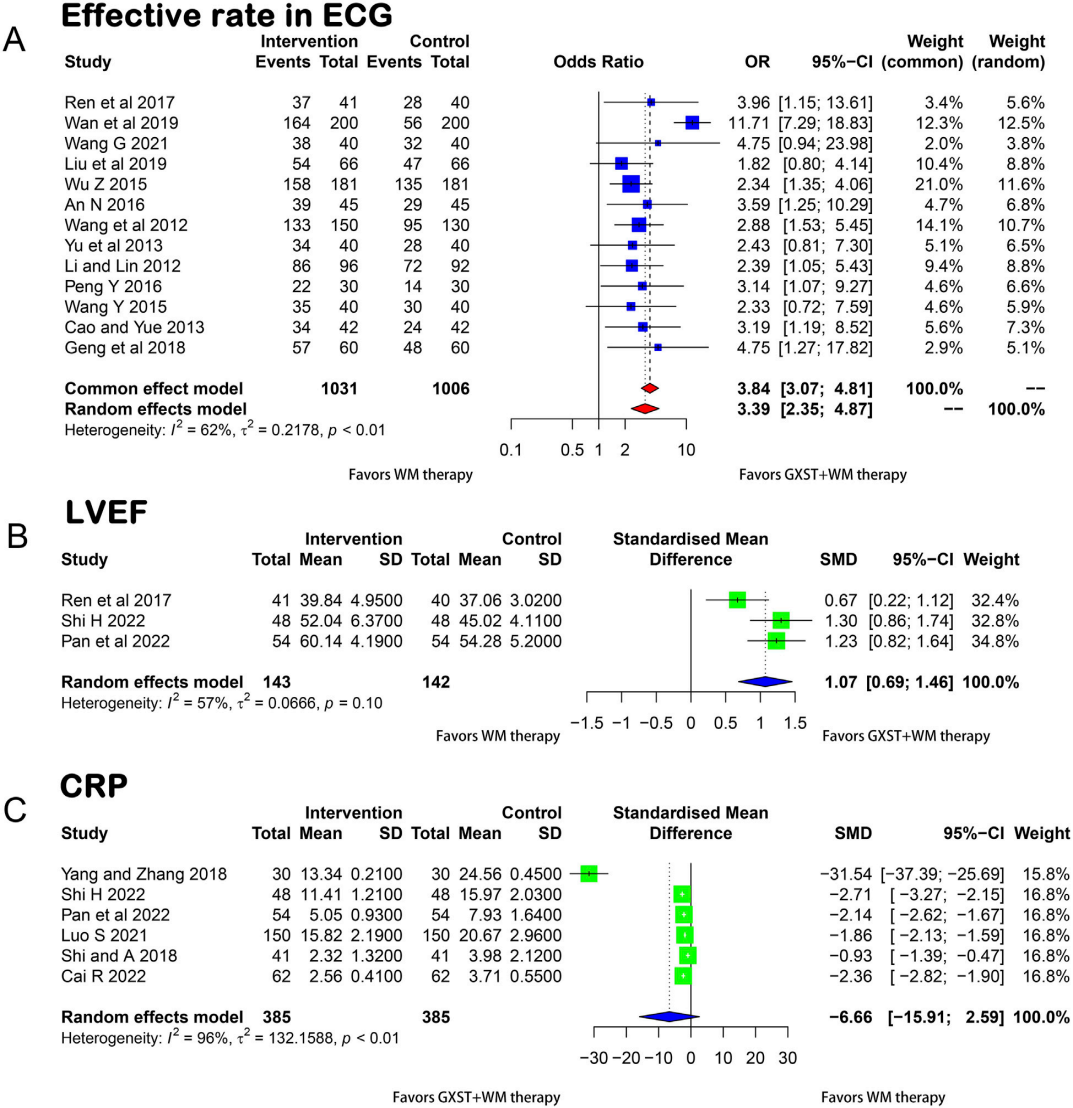
Forest plot for the meta-analysis **(A)** Effective rate in ECG; **(B)** LVEF; **(C)** CRP; GXST, Guanxinshutong; WM, Western medicine; ECG, Electrocardiogram; LVEF, Left Ventricular Ejection Fraction; CRP, C-reactive Protein.

Thirteen articles (Li and Li, 2012; Wang et al., 2012; Cao and Yue, 2013; Yu et al., 2013; Wang and Wu, 2015; Wu et al., 2015; An, 2016; Peng, 2016; Ren et al., 2017; Geng et al., 2018; Liu et al., 2019; Wan et al., 2019; Wang, 2021) reported ECG improvement rates. Due to heterogeneity among the trials (*p* < 0.01, I^2^ = 62%), a random-effects model was applied. The results showed that the ECG improvement rate was significantly better in the test group than in the control group (OR = 3.39, 95% CI = 2.35–4.87) and the difference was statistically significant (Figure 4A).

#### 3.3.2 Secondary outcome

##### 3.3.2.1 LVEF

Using a random-effects model (*p* = 0.1, I^2^ = 57%), three articles (Ren et al., 2017; Pan et al., 2022; Shi, 2022) presented the measurement of LVEF before and after treatment in the two groups. As illustrated in Figure 4B, the GXST and WM combination therapy significantly improved LVEF compared with WM therapy alone (MD = 1.07, 95% CI = 0.69–1.46).

##### 3.3.2.2 CRP

Six studies (Shi and Abudujilili, 2018; Yang and Zhang, 2018; Luo, 2021; Cai, 2022; Pan et al., 2022; Shi, 2022) compared CRP levels between the experimental and control groups. No significant difference was found between the two groups in terms of the reduction of CRP levels (*p* < 0.01, I^2^ = 96%, MD = −6.66, 95% CI = −15.91 to 2.59) (Figure 4C).

##### 3.3.2.3 TC

**FIGURE 5:**
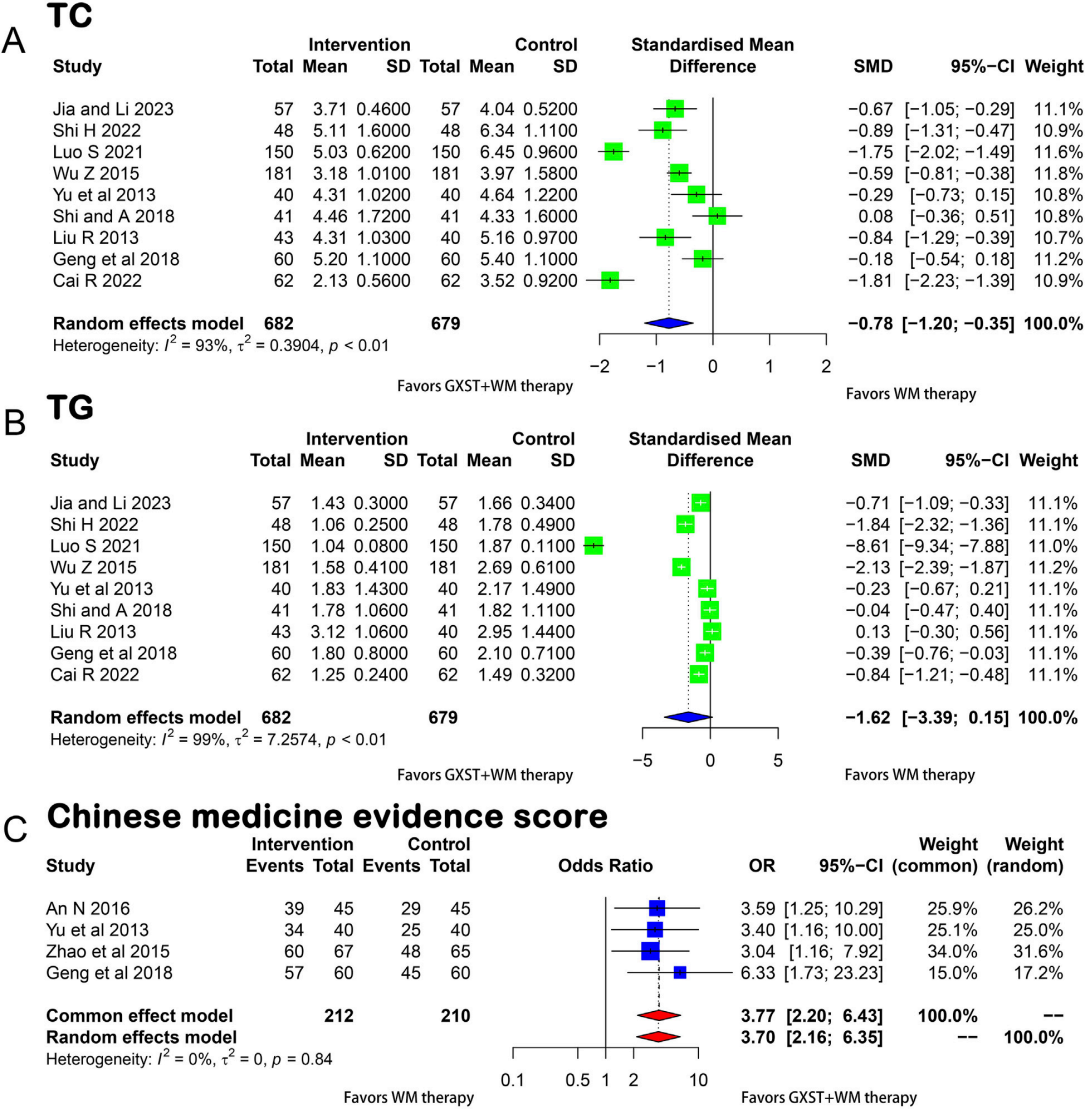
Forest plot for the meta-analysis **(A)** TC; **(B)** TG; **(C)** Chinese medicine evidence score; GXST, Guanxinshutong; WM, Western medicine; TC, Total Cholesterol; TG, Triglyceride.

Nine studies (Liu, 2013; Yu et al., 2013; Wu et al., 2015; Geng et al., 2018; Shi and Abudujilili, 2018; Luo, 2021; Cai, 2022; Shi, 2022; Jia and Li, 2023) compared the TC levels between the experimental and control groups. A meta-analysis of the five studies revealed that GXST and WM combination therapy significantly reduced TC levels in SAP patients (*p* < 0.01, I^2^ = 93%, MD = −0.78, 95% CI = −1.20 to −0.35) (Figure 5A).

##### 3.3.2.4 TG

Nine studies (Liu, 2013; Yu et al., 2013; Wu et al., 2015; Geng et al., 2018; Shi and Abudujilili, 2018; Luo, 2021; Cai, 2022; Shi, 2022; Jia and Li, 2023) reported TG levels. A meta-analysis conducted using a random-effects model (*p* < 0.01, I^2^ = 99%) showed no significant difference in the improvement of TG levels between the two groups (MD = −1.62, 95% CI = −3.39 to 0.15) (Figure 5B).

##### 3.3.2.5 Chinese medicine evidence score

Four RCTs (Yu et al., 2013; Zhao et al., 2015; An, 2016; Geng et al., 2018) reported Chinese medicine evidence score. Since homogeneity was found between the four studies (*p* = 0.84, I^2^ = 0), the fixed-effects model was utilized and the results demonstrated a significant improvement in the Chinese medicine evidence score in the experimental compared with the control group (OR = 3.77, 95% CI = 2.20–6.43) (Figure 5C). This score reflects the overall quality and effectiveness of the Chinese medicine treatment. The studies were homogeneous, indicating consistency in the methods and results. This was further confirmed by Labbé plots (Supplementary File S4).

##### 3.3.2.6 TNF-α

**FIGURE 6:**
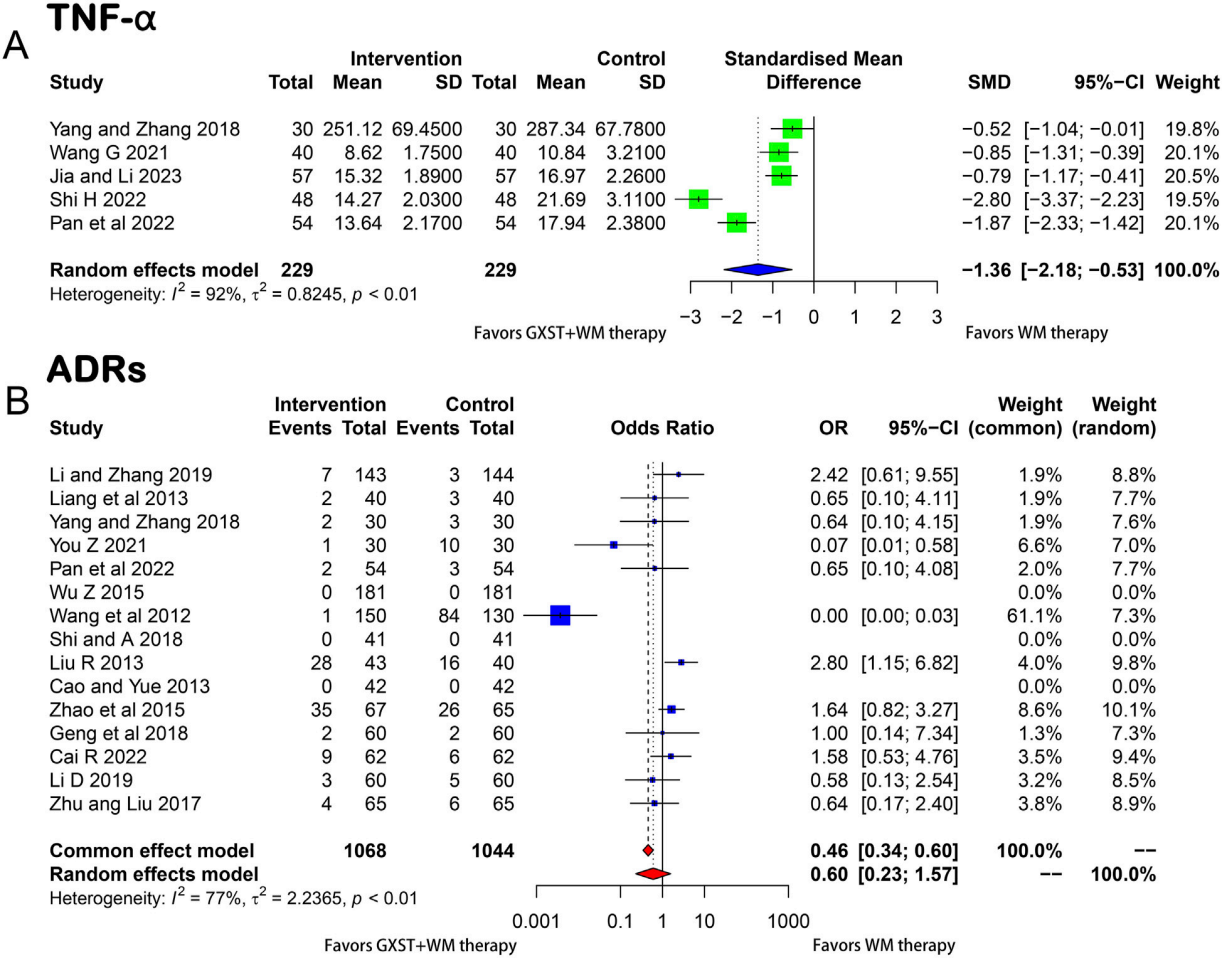
Forest plot for the meta-analysis **(A)**TNF-α; **(B)** ARDs; GXST, Guanxinshutong; WM, Western medicine; TNF-α, Tumor Necrosis Factor-alpha; ARDs, adverse medicine events.

A meta-analysis of five studies (Yang and Zhang, 2018; Wang, 2021; Pan et al., 2022; Shi, 2022; Jia and Li, 2023) showed that the GXST and WM combination therapy significantly reduced the TNF-α levels compared to WM therapy alone (*p* < 0.01, I^2^ = 92%, MD = −1.36, 95% CI = −2.18 to −0.53) (Figure 6A).

#### 3.3.3 ADRs

Fifteen studies (Wang et al., 2012; Cao and Yue, 2013; Liang et al., 2013; Liu, 2013; Wu et al., 2015; Zhao et al., 2015; Zhu and Liu, 2017; Geng et al., 2018; Shi and Abudujilili, 2018; Yang and Zhang, 2018; Li, 2019; Li et al., 2019; You, 2021; Cai, 2022; Pan et al., 2022) provided information on ADRs such as dizziness, nausea, and gastrointestinal bleeding. Due to heterogeneity between trials (*p* < 0.01, I^2^ = 77%), a random-effects model was used. The meta-analysis showed no significant difference in the incidence of ADRs between the two groups (OR = 0.60, 95% CI = 0.23–1.57) (Figure 6B).

#### 3.3.4 Analysis of publication bias

For outcomes comprising >10 studies, potential publication bias was explored using Egger’s and Begg’s tests, implemented through the “metabias” command. Egger’s test revealed no significant publication bias, using effective clinical rate as an example (Egger’s test: t = 0.23, *p* = 0.8206). However, the result of Begg’s test showed a potential publication bias (Begg’s test: z = 2.06, *p* = 0.0397). This suggests that studies with significant results or large effect sizes were more likely to be published, while those with non-significant results were overlooked. Considering that Egger’s test is based on a linear regression approach with more stringent assumptions about the distribution of the data and that the funnel plots show approximate symmetry (Supplementary File S5), it was concluded that there was no publication bias. Nevertheless, Begg’s test suggested a potential bias. Therefore, our conclusions should be interpreted with caution.

#### 3.3.5 Heterogeneity, meta-regression, and subgroup analyses

Meta-regression analyses were performed for all outcome indicators with I^2^ ≥ 50% and over five studies included. These analyses considered three covariates: treatment duration, medication dosage, and subject age. The choice of these covariates was based on their potential impact on the treatment outcomes. The results showed that subject age and treatment duration were correlated with the decline in TC and TG levels in both the experimental and control groups (Supplementary File S6). However, no significant correlation was observed between the three covariates and ECG, ADRs, or CRP levels in both groups. When subgroup analyses were performed for TC levels based on treatment duration, the relative heterogeneity within these subgroups was reduced, indicating that treatment duration was a significant contributor to the observed heterogeneity in the studies. Nevertheless, further investigation is needed to identify potential unmeasured covariates that may explain the remaining heterogeneity.

#### 3.3.6 Sensitivity analysis

For sensitivity analyses, each study was reviewed individually, and the original effective rate was specifically excluded in the ECG study that contained over five publications and demonstrated significant variability. The omission of a study by (Wan et al., 2019) led to a notable decrease in heterogeneity. Furthermore, there was an alteration in the OR and its 95% CI (Supplementary File S7). This evidence implies that the study could be the primary contributor to the detected heterogeneity.

## 4 Discussions

To the best of our knowledge, this is the first meta-analysis to explore the comprehensive efficacy of GXST and WM combination therapy in patients with SAP. The results indicated that the combination therapy significantly improved clinical efficacy, ECG, and symptoms of angina pectoris. Additionally, the study reveals that compared with the WM monotherapy group, the combined therapy group exhibited a significant increase in LVEF, a key determinant of cardiac function and prognosis in SAP patients. Moreover, the combination therapy significantly reduced TC and TNF-α levels but had no significant difference in the reduction of TG and CRP levels. Furthermore, the combined therapy alleviated symptoms such as chest tightness, palpitations, shortness of breath, and fatigue, thereby improving the scores of Chinese medicine evidence. It is noteworthy that no significant difference was found in the reduction of ADRs between the combined therapy and the WM monotherapy.

Based on its clinical manifestations and pathological characteristics, TCM has categorized stable coronary artery disease into “chest impediment,” “heart pain”, etc. (Yu et al., 2023) TCM posits that the pathogenesis of angina pectoris is characterized by “stagnation of Qi and blood,” where the impaired circulation of Qi and blood leads to localized stagnation of Qi and blood in the heart, forming an “impediment” that causes chest pain (Jin et al., 2021). GXST, with its efficacy in promoting blood circulation, removing blood stasis, and unblocking the channels, is the first new Mongolian medicine approved for clinical treatment of coronary heart disease angina pectoris (Li et al., 2019; Wang et al., 2021). In the formula, *S. miltiorrhiza* and Fructus Choerospondiatis primarily function to enhance Qi and activate blood circulation, while *Caryophyllus aromaticus* (Clove) and Borneol mainly serve to relieve pain. Bambusae Concretio Silicea plays a role in clearing heat and dissolving phlegm, which helps improve cardiac function and alleviate angina pectoris (Wang et al., 2024). Modern pharmacological studies have shown that the active metabolites in GXST can inhibit platelet aggregation and the release of inflammatory mediators, reduce blood viscosity, and improve hemorheology (Lu et al., 2020). This helps to reduce the cardiac burden and improve myocardial oxygen and blood supply conditions, thereby alleviating symptoms of angina pectoris. GXST can also lower blood lipids and inhibit the formation and development of atherosclerotic plaques, thereby playing a positive role in preventing the occurrence and progression of CVDs (Gao et al., 2021).

Dyslipidemia is an independent risk factor for CHD (Ariyanti and Besral, 2019). Research indicates that tanshinone, the primary active metabolite of *S. miltiorrhiza*, inhibits the activity of cholesterol synthesis enzymes in hepatocytes (such as 3-hydroxy-3-methylglutaryl coenzyme A [HMG-CoA] reductase), thereby reducing endogenous cholesterol production and effectively lowering lipid levels (Wresdiyati et al., 2023). Borneol can reduce lipid deposition in the body by promoting the oxidation and decomposition of fatty acids, thereby indirectly reducing lipid levels (Ran et al., 2021). Other metabolites in GXST, such as Fructus Choerospondiatis, Clove, and Bambusae Concretio Silicea, are also believed to reduce lipid levels by promoting fatty acid metabolism and inhibiting fat synthesis (Wang et al., 2024).

Inflammatory responses play a significant role in the pathophysiological mechanism of angina pectoris (Anzai, 2018). Tanshinone can inhibit the release of inflammatory mediators, such as TNF-α and interleukin-1 beta (IL-1β), by inflammatory cells (such as macrophages and neutrophils), alleviating the inflammatory response (Xu et al., 2022). It can also reduce tissue damage caused by inflammatory responses by scavenging free radicals and alleviating oxidative stress (Lu et al., 2022). Protocatechuic acid in Fructus Choerospondiatis can alleviate the inflammatory state during the development of atherosclerosis, protecting vascular endothelial cells (Zhang et al., 2021). Asiatic acid can reduce myocardial cell damage by inhibiting mitochondrial-dependent apoptosis and blocking TNF-α-mediated apoptosis (Zhang et al., 2022). It can also reduce the production of pro-inflammatory cytokines such as IL-1β and TNF-α (Legiawati et al., 2018). Quercetin has strong anti-lipid peroxidation activity and can exert anti-inflammatory effects by reducing the production of nitric oxide, inducible nitric oxide synthase, and IL-6 (Aminnezhad et al., 2023). Ellagic acid, gallic acid, and R-3,4-dihydroxyphenyl lactic acid (danshensu), three metabolites also reported in the literature to have antioxidant and anti-inflammatory biological activities, play a certain role in the treatment of SAP (Gupta et al., 2021). Stigmasterol can block the nuclear factor-kappa B (NF-κB) pathway to inhibit the expression of matrix metalloproteinases (MMPs) and the release of the pro-inflammatory mediator prostaglandin E2 (PGE2), exerting anti-inflammatory effects (Cai et al., 2023). Additionally, tanshinone and other components may reduce myocardial cell damage and protect myocardial function by inhibiting oxidative stress and inflammatory responses. Asiatic acid can reduce myocardial cell damage by inhibiting mitochondrial-dependent apoptosis and blocking TNF-α-mediated apoptosis (Zhang et al., 2022). It can also reduce the production of pro-inflammatory cytokines such as IL-1β and TNF-α (Legiawati et al., 2018). Quercetin has strong anti-lipid peroxidation activity and can exert anti-inflammatory effects by reducing the production of nitric oxide, inducible nitric oxide synthase, and IL-6 (Aminnezhad et al., 2023).

Several meta-analyses (Sui et al., 2016; Jia and Wei, 2017) have evaluated the efficacy of GXST and WM combination therapy for the treatment of angina pectoris and found that this combination therapy can effectively alleviate symptoms of angina pectoris, improve ECGs, and reduce the occurrence of ADRs. However, the scope of these studies is relatively narrow. Moreover, the lack of sensitivity or meta-regression analysis to systematically analyze the sources of heterogeneity reduces the reliability and validity of the results. Therefore, the current study comprehensively evaluated the efficacy and safety of the GXST and WM combination therapy for the treatment of patients with SAP in terms of clinical efficacy, ECG, LVEF, levels of TC, TG, CRP, TNF-α, Chinese medicine evidence score, and ADRs. Additionally, we conducted meta-regression and sensitivity subgroup analyses to identify and analyze the sources of heterogeneity, ensuring the robustness of the results.

Despite the encouraging results, our study has some limitations. Chiefly, studies' Chinese focus limits generalizability, and methodological issues compromise reliability. Future studies should adopt rigorous designs and cover broader regions. Heterogeneity in outcome measures highlights the need for standardization. Long-term safety, sustainability, and cost-effectiveness remain unaddressed due to short follow-ups. Assessing publication bias and exploring diverse patient populations are crucial to strengthen the evidence base. In conclusion, while our findings are insightful, addressing these limitations through rigorous, long-term, and comprehensive studies is necessary to establish a stronger evidence foundation.

Therefore, the current study results should be interpreted with caution. We believe that future large-scale RCTs using standardized intervention protocols will help obtain more reliable evidence. Moreover, a strict selection of studies to ensure study quality and relevance, ensuring methodological rigor, enhancing the objectivity of interpretations, and improving methodological quality will also boost the quality of future research.

## 5 Conclusion

In summary, the present study demonstrates that the GXST and WM combination therapy can improve clinical efficacy in SAP patients. However, this conclusion warrants further validation before the combination therapy can be implemented in clinical practice.
