# Supplementary material for: Efficacy and safety of guanxinshutong capsule combined with western medicine on stable angina pectoris: a systematic review and meta-analysis
Source: Front Pharmacol. 2024 Oct 30;15:1444388. doi: 10.3389/fphar.2024.1444388 (PMC11557469; doi:10.3389/fphar.2024.1444388)
Supplement: Supplementary file 1 [file Table4.DOC]

**Supplementary File S6**.Meta-Regression Analyses

| **EGG** |  | estimate | se | zval | pval | ci.lb | ci.ub |
| --- | --- | --- | --- | --- | --- | --- | --- |
|  | intrcpt | 1.4141 | 1.0706 | 1.3209 | 0.1865 | -0.6841 | 3.5124 |
|  | Dose 0.6g tid | 1.0467 | 1.0976 | 0.9536 | 0.3403 | -1.1047 | 3.198 |
|  | Dose 0.9g tid | -0.4412 | 0.8717 | -0.5061 | 0.6128 | -2.1497 | 1.2673 |
|  | Time 32 weeks | 0.49 | 0.8433 | 0.5811 | 0.5612 | -1.1629 | 2.1429 |
|  | Time 4 weeks | 0.144 | 0.6808 | 0.2115 | 0.8325 | -1.1904 | 1.4784 |
|  | Time 6 weeks | -0.0584 | 0.6011 | -0.0971 | 0.9226 | -1.2365 | 1.1198 |
|  | Time 8 weeks | 0.5852 | 0.9979 | 0.5864 | 0.5576 | -1.3707 | 2.5411 |
|  | TimeNR | -0.1021 | 0.8464 | -0.1207 | 0.9039 | -1.761 | 1.5567 |
| **ARDs** | intrcpt | -1.9668 | 3.1422 | -0.6259 | 0.5314 | -8.1253 | 4.1918 |
|  | Dose 0.9g tid | -0.0097 | 3.1305 | -0.0031 | 0.9975 | -6.1454 | 6.126 |
|  | Time4 weeks | 0.9024 | 3.0377 | 0.297 | 0.7664 | -5.0515 | 6.8562 |
|  | Time6 weeks | 1.4715 | 3.0199 | 0.4873 | 0.6261 | -4.4475 | 7.3904 |
|  | Age≥60 | 1.5346 | 2.2323 | 0.6875 | 0.4918 | -2.8405 | 5.9098 |
|  | AgeNR | 1.9765 | 3.864 | 0.5115 | 0.609 | -5.5969 | 9.5498 |
| **TC** | intrcpt | -0.3041 | 0.3469 | -0.8767 | 0.3806 | -0.9841 | 0.3758 |
|  | Time 2 weeks | 0.0135 | 0.6073 | 0.0222 | 0.9823 | -1.1767 | 1.2037 |
|  | Time 4 weeks | -1.0459 | 0.4922 | -2.1249 | 0.0336 | -2.0105 | -0.0812 |
|  | Time 6 weeks | -0.4025 | 0.4842 | -0.8313 | 0.4058 | -1.3515 | 0.5465 |
|  | Time 8 weeks | -1.4487 | 0.5803 | -2.4965 | 0.0125 | -2.586 | -0.3113 |
|  | AgeNR | 1.5722 | 0.6691 | 2.3496 | 0.0188 | 0.2607 | 2.8836 |
| **TG** | intrcpt | -0.3763 | 0.7445 | -0.5054 | 0.6133 | -1.8354 | 1.0828 |
|  | Time 2 weeks | 0.1457 | 1.2923 | 0.1127 | 0.9102 | -2.3871 | 2.6784 |
|  | Time 4 weeks | -0.958 | 1.0539 | -0.909 | 0.3633 | -3.0235 | 1.1075 |
|  | Time 6 weeks | -0.6384 | 1.0503 | -0.6078 | 0.5433 | -2.697 | 1.4202 |
|  | Time 8 weeks | -8.2319 | 1.3259 | -6.2086 | <.0001 | -10.8306 | -5.6332 |
|  | AgeNR | 8.2141 | 1.5176 | 5.4125 | <.0001 | 5.2396 | 11.1885 |
| **CRP** | intrcpt | -0.9312 | 10.9001 | -0.0854 | 0.9319 | -22.295 | 20.4326 |
|  | Time4 weeks | 6.982 | 14.2964 | 0.4884 | 0.6253 | -21.0384 | 35.0023 |
|  | Time8 weeks | -9.1199 | 14.2957 | -0.6379 | 0.5235 | -37.1391 | 18.8992 |
|  | Age≥60 | -12.6805 | 10.17 | -1.2469 | 0.2125 | -32.6133 | 7.2523 |
