# Supplementary figures and images for: Efficacy and safety of guanxinshutong capsule combined with western medicine on stable angina pectoris: a systematic review and meta-analysis
Source: Front Pharmacol. 2024 Oct 30;15:1444388. doi: 10.3389/fphar.2024.1444388 (PMC11557469; doi:10.3389/fphar.2024.1444388)

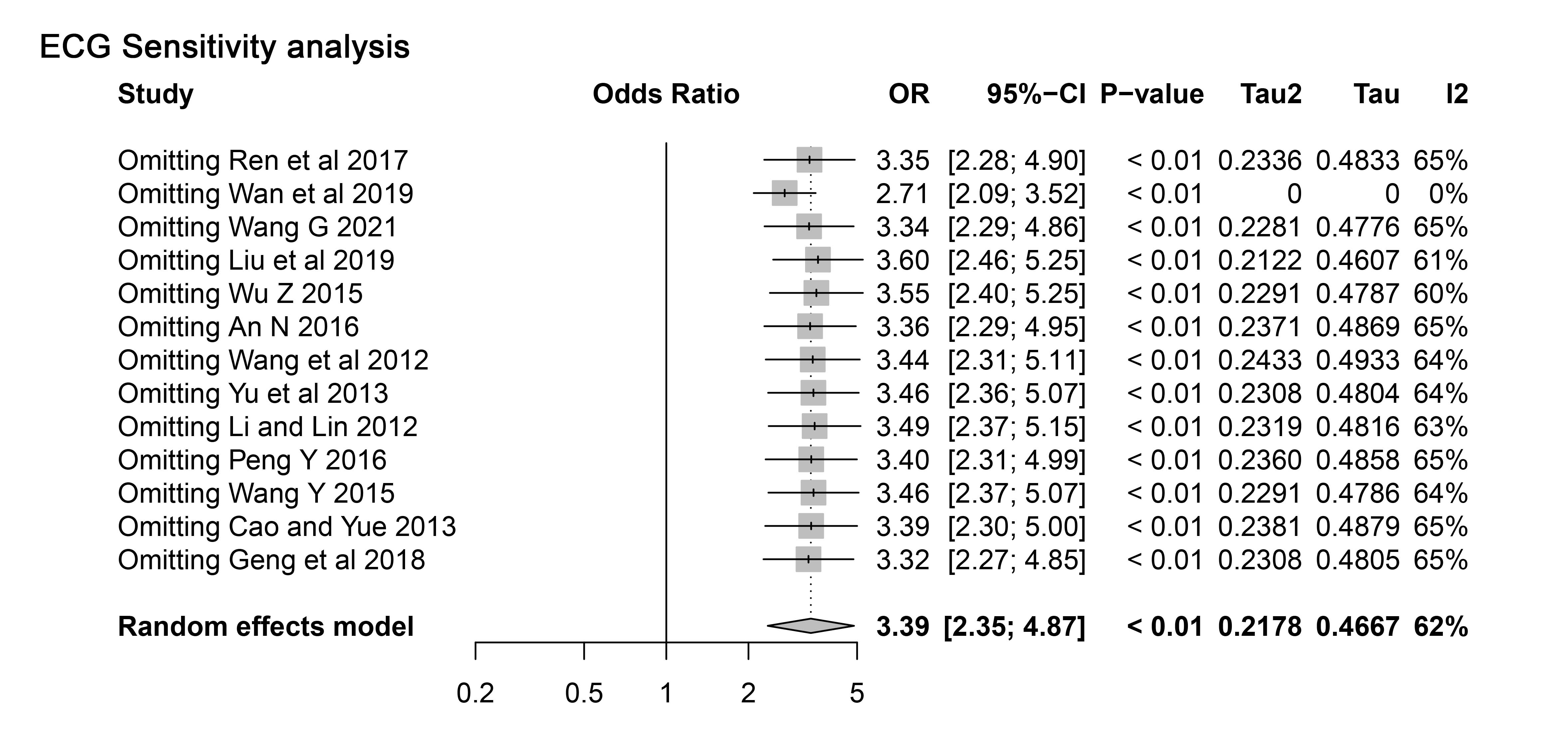

Supplement: Supplementary file 2 [file Image3.JPEG]

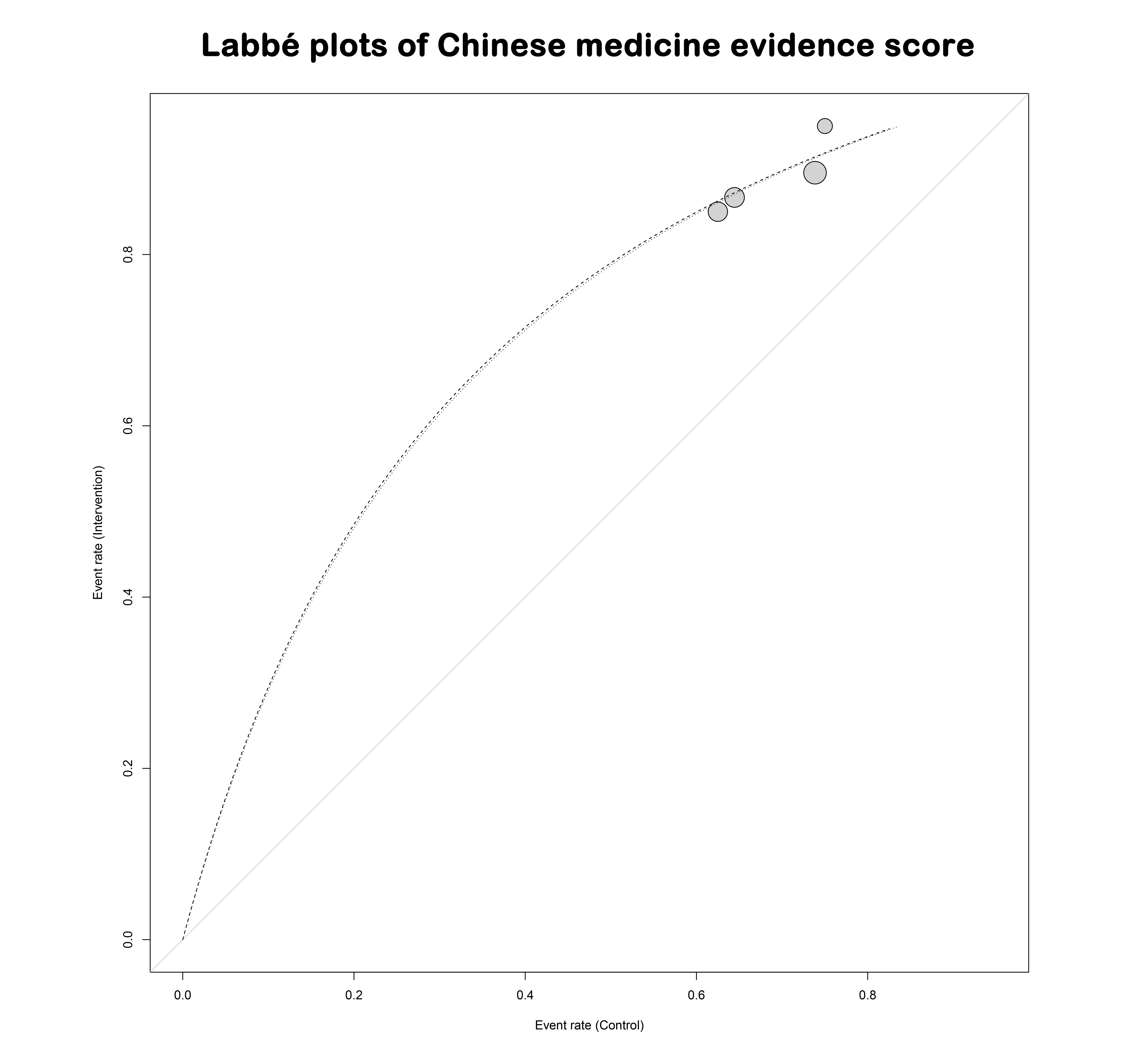

Supplement: Supplementary file 4 [file Image1.JPEG]

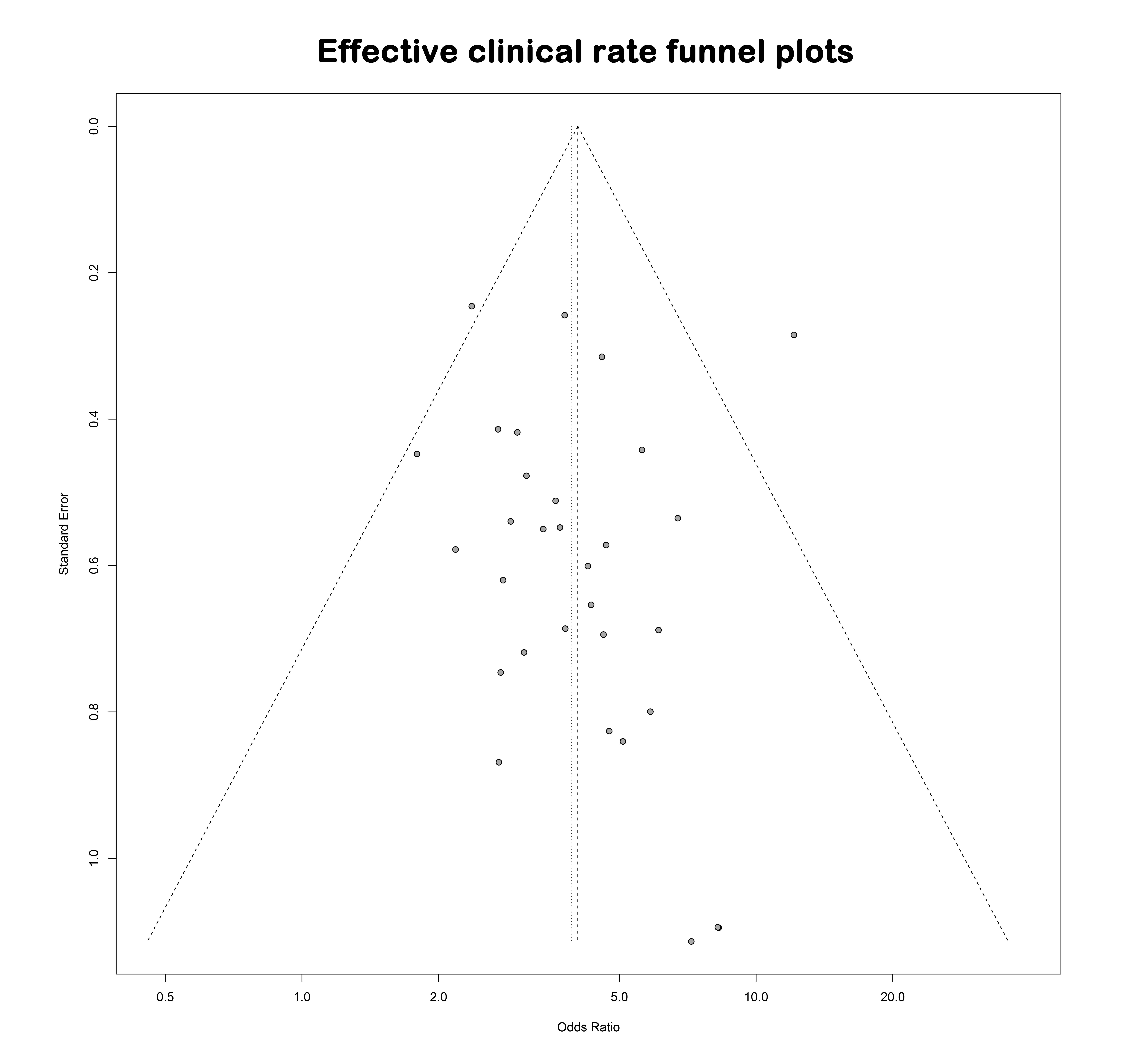

Supplement: Supplementary file 5 [file Image2.JPEG]
