# Supplementary material for: Efficacy and safety of guanxinshutong capsule combined with western medicine on stable angina pectoris: a systematic review and meta-analysis
Source: Front Pharmacol. 2024 Oct 30;15:1444388. doi: 10.3389/fphar.2024.1444388 (PMC11557469; doi:10.3389/fphar.2024.1444388)
Supplement: Supplementary file 3 [file Table3.DOC]

# Supplementary File S3 ：Search strategy for meta-analysis.

# Search strategy of China National Knowledge Infrastructure.

| No. | Search items |
| --- | --- |
| #1 | SU %= '冠心病' OR SU %= '心绞痛' OR SU %= '稳定型心绞痛' |
| #2 | SU %= '冠心舒通' |
| #3 | FT = '随机' |
| #4 | #1 AND #2 AND #3 |

## Search strategy of Wanfang Database.

| No. | Search items |
| --- | --- |
| #1 | 主题:(冠心病) or 主题:(心绞痛) or 主题:(稳定型心绞痛) |
| #2 | 主题:(冠心舒通) |
| #3 | 全部:(随机) |
| #4 | #1 AND #2 AND #3 |

## Search strategy of Chinese Biomedical Literature Database.

| No. | Search items |
| --- | --- |
| #1 | "冠心病"[常用字段:智能] OR "心绞痛"[常用字段:智能] OR "稳定型心绞痛"[常用字段:智能] |
| #2 | "冠心舒通"[常用字段:智能] |
| #3 | "随机"[全部字段:智能]) |
| #4 | #1 AND #2 AND #3 |

## Search strategy of Weipu Journal Database.

| No. | Search items |
| --- | --- |
| #1 | M=(冠心病 OR 心绞痛 OR 稳定型心绞痛） |
| #2 | M=(冠心舒通) |
| #3 | U=(随机) |
| #4 | #1 AND #2 AND #3 |

## Search strategy of Pubmed.

| No. | Search items |
| --- | --- |
| #1 | " Angina Pectoris"[MeSH Terms] |
| #2 | "Prinzmetal's Angina"[Title/Abstract] OR "Angina, Prinzmetal's"[Title/Abstract] OR "Prinzmetals Angina"[Title/Abstract] OR "Prinzmetal Angina"[Title/Abstract] OR "Angina, Prinzmetal"[Title/Abstract] |
| #3 | #1 OR #2 |
| #4 | "guanxinshutong"[Title/Abstract] |
| #5 | "controlled clinical trial"[Publication Type] OR "randomized controlled trial"[Publication Type] OR "equivalence trial"[Publication Type] OR "pragmatic clinical trial"[Publication Type] OR "random*"[All Fields] |
| #6 | #3 AND #4 AND #5 |

## Search strategy of Embase.

| No. | Search items |
| --- | --- |
| #1 | 'Angina Pectoris'/exp OR 'Prinzmetal's Angina':ti,ab,kw OR 'angioplasty, transluminal coronary':ti,ab,kw OR 'angioplasty, transluminal, percutaneous coronary' :ti,ab,kw OR 'coronary angioplasty':ti,ab,kw OR 'coronary angioplasty, transluminal' :ti,ab,kw OR 'coronary artery dilatation, transluminal':ti,ab,kw OR 'coronary balloon angioplasty':ti,ab,kw OR 'p.t.c.a.':ti,ab,kw OR 'percutaneous coronary transluminal angioplasty':ti,ab,kw OR 'percutaneous transluminal coronary angioplasty' :ti,ab,kw OR 'ptca':ti,ab,kw |
| #2 | guanxinshutong:ti,ab,kw |
| #3 | 'randomized controlled trial'/exp OR 'equivalence trial'/exp OR 'non-inferiority trial'/exp OR 'pragmatic trial'/exp OR 'superiority trial'/exp OR 'controlled clinical trial':it OR 'randomized controlled trial':it OR 'equivalence trial':it OR 'pragmatic clinical trial':it OR 'superiority trial':it OR 'non-inferiority trial':it OR random* |
| #4 | #1 AND #2 AND #3 |

## Search strategy of Cochrane Library.

| No. | Search items |
| --- | --- |
| #1 | MeSH descriptor: [Angina Pectoris] explode all trees |
| #2 | (Prinzmetal's Angina; or Angina, Prinzmetal's; or Prinzmetals Angina; or Prinzmetal Angina; or Angina, Prinzmetal):ti,ab,kw |
| #3 | #1 OR #2 |
| #4 | (guanxinshutong):ti,ab,kw |
| #5 | MeSH descriptor: [Randomized Controlled Trial] explode all trees |
| #6 | (Randomized Controlled Trial):pt OR (Controlled Clinical Trial):pt OR (Equivalence Trial):pt OR (Pragmatic Clinical Trial):pt OR (random*) |
| #7 | #5 OR #6 |
| #8 | #3 AND #4 AND #7 |

# Supplementary File S2 ：Search strategy for meta-analysis.

# Search strategy of China National Knowledge Infrastructure.

| No. | Search items |
| --- | --- |
| #1 | SU %= 'Coronary Heart Disease' OR SU %= ' Angina Pectoris' OR SU %= ' Stable Angina Pectoris' |
| #2 | SU %= 'Guanxinshutong' |
| #3 | FT = 'Random' |
| #4 | #1 AND #2 AND #3 |

## Search strategy of Wanfang Database.

| No. | Search items |
| --- | --- |
| #1 | Topic:(Angina Pectoris) or Topic:(Coronary Heart Disease) Topic:(Stable Angina Pectoris) |
| #2 | Topic:(Guanxinshutong) |
| #3 | All:(Random) |
| #4 | #1 AND #2 AND #3 |

## Search strategy of Chinese Biomedical Literature Database.

| No. | Search items |
| --- | --- |
| #1 | "Coronary Heart Disease"[Common field: Intelligence] OR "Angina Pectoris"[Common field: Intelligence] OR "Stable Angina Pectoris"[Common field: Intelligence] |
| #2 | "Guanxinshutong"[Common field: Intelligence] |
| #3 | "Random"[All fields: Intelligence]) |
| #4 | #1 AND #2 AND #3 |

## Search strategy of Weipu Journal Database.

| No. | Search items |
| --- | --- |
| #1 | M=(Coronary Heart Disease OR Angina Pectoris OR Stable Angina Pectoris) |
| #2 | M=(Guanxinshutong） |
| #3 | U=(Random) |
| #4 | #1 AND #2 AND #3 |

## Search strategy of Pubmed.

| No. | Search items |
| --- | --- |
| #1 | " Angina Pectoris"[MeSH Terms] |
| #2 | "Prinzmetal's Angina"[Title/Abstract] OR "Angina, Prinzmetal's"[Title/Abstract] OR "Prinzmetals Angina"[Title/Abstract] OR "Prinzmetal Angina"[Title/Abstract] OR "Angina, Prinzmetal"[Title/Abstract] |
| #3 | #1 OR #2 |
| #4 | "guanxinshutong"[Title/Abstract] |
| #5 | "controlled clinical trial"[Publication Type] OR "randomized controlled trial"[Publication Type] OR "equivalence trial"[Publication Type] OR "pragmatic clinical trial"[Publication Type] OR "random*"[All Fields] |
| #6 | #3 AND #4 AND #5 |

## Search strategy of Embase.

| No. | Search items |
| --- | --- |
| #1 | 'Angina Pectoris'/exp OR 'Prinzmetal's Angina':ti,ab,kw OR 'angioplasty, transluminal coronary':ti,ab,kw OR 'angioplasty, transluminal, percutaneous coronary' :ti,ab,kw OR 'coronary angioplasty':ti,ab,kw OR 'coronary angioplasty, transluminal' :ti,ab,kw OR 'coronary artery dilatation, transluminal':ti,ab,kw OR 'coronary balloon angioplasty':ti,ab,kw OR 'p.t.c.a.':ti,ab,kw OR 'percutaneous coronary transluminal angioplasty':ti,ab,kw OR 'percutaneous transluminal coronary angioplasty' :ti,ab,kw OR 'ptca':ti,ab,kw |
| #2 | guanxinshutong:ti,ab,kw |
| #3 | 'randomized controlled trial'/exp OR 'equivalence trial'/exp OR 'non-inferiority trial'/exp OR 'pragmatic trial'/exp OR 'superiority trial'/exp OR 'controlled clinical trial':it OR 'randomized controlled trial':it OR 'equivalence trial':it OR 'pragmatic clinical trial':it OR 'superiority trial':it OR 'non-inferiority trial':it OR random* |
| #4 | #1 AND #2 AND #3 |

## Search strategy of Cochrane Library.

| No. | Search items |
| --- | --- |
| #1 | MeSH descriptor: [Angina Pectoris] explode all trees |
| #2 | (Prinzmetal's Angina; or Angina, Prinzmetal's; or Prinzmetals Angina; or Prinzmetal Angina; or Angina, Prinzmetal):ti,ab,kw |
| #3 | #1 OR #2 |
| #4 | (guanxinshutong):ti,ab,kw |
| #5 | MeSH descriptor: [Randomized Controlled Trial] explode all trees |
| #6 | (Randomized Controlled Trial):pt OR (Controlled Clinical Trial):pt OR (Equivalence Trial):pt OR (Pragmatic Clinical Trial):pt OR (random*) |
| #7 | #5 OR #6 |
| #8 | #3 AND #4 AND #7 |
