# Supplementary material for: Efficacy and safety of guanxinshutong capsule combined with western medicine on stable angina pectoris: a systematic review and meta-analysis
Source: Front Pharmacol. 2024 Oct 30;15:1444388. doi: 10.3389/fphar.2024.1444388 (PMC11557469; doi:10.3389/fphar.2024.1444388)
Supplement: Supplementary file 6 [file Table1.DOC]

**Supplementary File S1.** （**table of the main components of Guanxinshutong capsule**）

| **English/Latin Name** | **Chinese Name** | **Family** | **Species** | **Molecule ID** | **Molecule** |
| --- | --- | --- | --- | --- | --- |
| Salviae miltiorrhizae radix et rhizoma / Salvia miltiorrhiza Bunge | Danshen | Lamiaceae | S. miltiorrhiza | MOL001659 | Poriferasterol |
|  |  |  |  | MOL002651 | Dehydrotanshinone II A |
|  |  |  |  | MOL007041 | 2-isopropyl-8-methylphenanthrene-3,4-dione |
|  |  |  |  | MOL007045 | 3α-hydroxytanshinoneⅡa |
|  |  |  |  | MOL007048 | (E)-3-[2-(3,4-dihydroxyphenyl)-7-hydroxy-benzofuran-4-yl]acrylic acid |
|  |  |  |  | MOL007050 | 2-(4-hydroxy-3-methoxyphenyl)-5-(3-hydroxypropyl)-7-methoxy-3-benzofurancarboxaldehyde |
|  |  |  |  | MOL007058 | formyltanshinone |
|  |  |  |  | MOL007068 | Przewaquinone B |
|  |  |  |  | MOL007069 | przewaquinone c |
|  |  |  |  | MOL007070 | (6S,7R)-6,7-dihydroxy-1,6-dimethyl-8,9-dihydro-7H-naphtho[8,7-g]benzofuran-10,11-dione |
|  |  |  |  | MOL007071 | przewaquinone f |
|  |  |  |  | MOL007077 | sclareol |
|  |  |  |  | MOL007079 | tanshinaldehyde |
|  |  |  |  | MOL007081 | Danshenol B |
|  |  |  |  | MOL007082 | Danshenol A |
|  |  |  |  | MOL007088 | cryptotanshinone |
|  |  |  |  | MOL007094 | danshenspiroketallactone |
|  |  |  |  | MOL007098 | deoxyneocryptotanshinone |
|  |  |  |  | MOL007101 | dihydrotanshinoneⅠ |
|  |  |  |  | MOL007108 | isocryptotanshi-none |
|  |  |  |  | MOL007111 | Isotanshinone II |
|  |  |  |  | MOL007115 | manool |
|  |  |  |  | MOL007119 | miltionone Ⅰ |
|  |  |  |  | MOL007125 | neocryptotanshinone |
|  |  |  |  | MOL007130 | prolithospermic acid |
|  |  |  |  | MOL007140 | (Z)-3-[2-[(E)-2-(3,4-dihydroxyphenyl)vinyl]-3,4-dihydroxy-phenyl]acrylic acid |
|  |  |  |  | MOL007150 | (6S)-6-hydroxy-1-methyl-6-methylol-8,9-dihydro-7H-naphtho[8,7-g]benzofuran-10,11-quinone |
|  |  |  |  | MOL007151 | Tanshindiol B |
|  |  |  |  | MOL007152 | Przewaquinone E |
|  |  |  |  | MOL007154 | tanshinone iia |
|  |  |  |  | MOL007155 | (6S)-6-(hydroxymethyl)-1,6-dimethyl-8,9-dihydro-7H-naphtho[8,7-g]benzofuran-10,11-dione |
|  |  |  |  | MOL007156 | tanshinone Ⅵ |
| Choerospondiatis Fructus / Choerospondias axillaris (Roxb.) | Guangzao | Anacardiaceae | Choerospondias axillaris | MOL001040 | (2R)-5,7-dihydroxy-2-(4-hydroxyphenyl)chroman-4-one |
|  |  |  |  | MOL001736 | (-)-taxifolin |
|  |  |  |  | MOL000422 | kaempferol |
|  |  |  |  | MOL004328 | naringenin |
|  |  |  |  | MOL000098 | quercetin |
| Caryophylliflos / Syzygium aromaticum （L.） | Dingxiang | Myrtaceae | Syzygium aromaticum | MOL013219 | Strictosamide_qt |
|  |  |  |  | MOL000422 | kaempferol |
|  |  |  |  | MOL000449 | Stigmasterol |
|  |  |  |  | MOL000098 | quercetin |
| Borneolum / Cinnamomum camphora（L.）Presl | Bingpian | Lauraceae | Camphora officinarum | MOL006861 | asiatic acid |
|  |  |  |  | MOL006862 | bronyl acetate |
|  |  |  |  | MOL006865 | dipterocarpol |
| Bambusae Concretio Silicea / Cephalostachyum chinense (Rendle) | Tianzhuhuang | Poaceae | Cephalostachyum chinense | NR | NR |

**Note:** The complex compounds of 5 botanical drugs of GXST were obtained from TCMSP (<http://tcmspw.com/tcmsp.php>), which is the largest noncommercial TCM database worldwide. TCMSP have collected all the 499 herbs registered in Chinese pharmacopoeia (2010), with a total of 12144 chemical（https://tcmspe.com/load_intro.php?id=40）
